# Differential CD4 T Regulatory Cell Phenotype Induced by Andes Hantavirus Glycoprotein

**DOI:** 10.3389/fcimb.2020.00430

**Published:** 2020-08-25

**Authors:** Farides Saavedra, Jose L. Garrido, Francisco Fuentes-Villalobos, Mario Calvo, Raúl Riquelme, María Luisa Rioseco, Carolina Chahín, Leonila Ferreira, Raymond Alvarez, Estefania Nova-Lamperti, Maria Ines Barria

**Affiliations:** ^1^Department of Microbiology, Faculty of Biological Science, Biotechnology Center, Universidad de Concepción, Concepción, Chile; ^2^Ichor Biologics LLC, New York, NY, United States; ^3^Institute of Medicine, Universidad Austral de Chile, Valdivia, Chile; ^4^Hospital Puerto Montt Dr. Eduardo Schoütz Schroeder, Puerto Montt, Chile; ^5^Hospital Regional Temuco Dr. Hernán Henríquez Aravena, Temuco, Chile; ^6^Hospital Clínico Regional Guillermo Grant Benavente, Concepción, Chile; ^7^Department of Clinical Biochemistry and Immunology, Faculty of Pharmacy, Universidad de Concepción, Concepción, Chile

**Keywords:** CD4 Treg, ANDV-GP, VLPs, HCPS, Th-like Treg, CXCR3, Th1/Th2

## Abstract

Hantavirus cardiopulmonary syndrome (HCPS) caused by Andes orthohantavirus (ANDV) in South America is a public health threat due to the significant rate of mortality and the lack of a specific treatment. Interestingly, the virus does not produce cytopathic effect, thereby the strong antiviral immune response is suspected to contribute to pathogenesis, hence is important to understand the balance between protective and harmfully immunity. CD4^+^ T regulatory cells (Treg) are essential to control an exacerbated immune response. In human ANDV infection, little is known about CD4^+^ Treg cells, which may be involved in control immunopathology associated to the infection. In this report, we characterize the phenotype of memory CD4^+^ Tregs in a HCPS survivor's cohort. Based on the expression of CXCR3, CCR4, and CCR6, we identified different Th-like Treg populations in ANDV survival's PBMCs. In addition, the effect of ANDV-glycoprotein virus like particles (VLP) was determined. We demonstrated that memory CD4^+^ Treg from HCPS present a specific phenotype, showing higher frequency of PD-1 compared to healthy donors (HD). In addition, it was observed a decrease in the frequency of Th1-like memory CD4^+^ Treg in HCPS, important to highlight that this signature could be preserved even years after resolution of infection. Moreover, to gain insight in the mechanism involved, we evaluated whether ANDV-glycoprotein (GP) VLP could modulate CD4^+^ Treg. Interestingly, ANDV-GP VLP induced a decrease in the frequency of CXCR3 (Th1-like) and an increase in CCR4 (Th2-like) memory CD4^+^ Treg in both HD and HCPS PBMCs, indicating that ANDV-GP could specifically act over CXCR3 and CCR4 in CD4^+^ Treg. This report contributes to the study of human CD4^+^ Treg cells in ANDV infection.

## Introduction

Andes orthohantavirus (ANDV) is an important emerging zoonotic pathogen, and one of the most significant causative agent of hantavirus cardiopulmonary syndrome (HCPS) in South America, with a high rate of mortality (Hjelle and Torres-Perez, 2010). In ANDV infection the clinical course is variable ranging from moderate diseases to fatal outcome in around 30–40% of HCPS cases (Jonsson et al., 2010). Although differences in the virus strain might affect the outcome, it is more likely that host factors are important in determining the immune response established to neutralize the virus and generate the clearance of infected cells (Hjelle and Torres-Perez, 2010; Vaheri et al., 2013).

The primary infection is produced through the inhalation of aerosolized viral particles present in the urine and feces of infected rodents. The virus enters through the respiratory epithelium and vascular endothelial cells are the main primary targets of hantavirus infection resulting in a capillary leak syndrome and edema (Sundstrom et al., 2001; Taylor et al., 2013; Figueiredo et al., 2014). Interestingly, the virus does not produce cytopathic effect (Mackow and Gavrilovskaya, 2009).

One of the hallmarks of the pathology shown by acute HCPS patients is the intense immune response characterized by CD8^+^ T cells infiltration (Kilpatrick et al., 2004; Terajima et al., 2007; Wang et al., 2009) and the cytokine storm produced during acute HCPS (Morzunov et al., 2015; Angulo et al., 2017; Maleki et al., 2019). Because the immunopathology is thought to be important during HCPS and the balance between protective and harmfully immune response is unknown, CD4^+^ T regulatory (Treg) response could play an important role during and to resolve infection (Schountz et al., 2007; Lindgren et al., 2011).

In HCPS, the intense antiviral immune response is suspected of contributing to pathogenesis. In fact, Th1-derived IFNγ cells have been associated to severe disease and unfavorable outcomes where cytotoxic CD8^+^ T cells target infected endothelium cells making this action critical to develop respiratory distress (Rasmuson et al., 2016). Importantly, mononuclear cell infiltrates are found in autopsy lung specimens from HCPS patients, and many of these cells secrete proinflammatory cytokines, including TNFα, lymphotoxin (LT), IL-2, IL-4, and IFNγ (Mori et al., 1999). CD4^+^ and cytotoxic CD8^+^ T cells have been isolated from Sin Nombre virus (SNV) infected patients, and a prominent role of CD8^+^ T cells have been suggested to be involved in the severity of HCPS (Ennis et al., 1997; Kilpatrick et al., 2004).

Currently, there is little knowledge in the specific role of CD4^+^ T subpopulations in HCPS disease. Between them, CD4^+^ Tregs are crucial in reducing an exacerbated immune response and limit chronic infection (Vignali et al., 2008). CD4^+^ T cells with a regulatory phenotype were defined as FoxP3^+^ (Fontenot et al., 2003; Hori et al., 2003) that expressed high levels of CD25 and lacked expression of CD127 (Sakaguchi et al., 1995; Liu et al., 2006; Hartigan-O'Connor et al., 2007). In addition, different memory Treg populations have been described that differ in expression of pro and anti-inflammatory cytokines and that mirror the CD4^+^ T helper (Th) cell phenotypes based on the expression of chemokine receptors CXCR3, CCR4, and CCR6, expressed by T-bet (Th1), GATA3 (Th2), and RORγt (Th17) cells, respectively, which were describe as Th1-like Treg, Th2-like Treg, and Th17-like Treg (Duhen et al., 2012; Halim et al., 2017).

Regarding CD4^+^ Treg response in hantavirus, previous study showed that, the frequencies of circulating CD4^+^ Treg cells remain unchanged in Puumala hantavirus (PUUV) infection (Lindgren et al., 2011). However, other T cell regulatory mechanisms, such as the expression of the inhibitory receptors cytotoxic T-lymphocyte antigen 4 (CTLA-4) and programmed death 1 protein (PD-1) as well as high levels of regulatory cytokines (Green et al., 1999; Hutchinson and Rollin, 2007) would exert a regulatory role in viral infectious diseases (Veiga-Parga et al., 2013; de Alwis et al., 2016; Ruibal et al., 2016; Schonrich and Raftery, 2019), as well as in limiting lung inflammation in respiratory infections (Fulton et al., 2010).

With the goal to identify markers in the memory CD4^+^ T cell compartment that may give us insights in the immunopathology associated to ANDV infection, in this study we have examined the CD4^+^ Treg cell profile in HCPS survivors of ANDV infection. We also assessed the phenotype of CD4^+^ Treg cells stimulated with ANDV-glycoproteins (GP) viral-like particles (VLPs) as a potential modulator of CD4^+^ Treg response. Moreover, based on the expression of CXCR3, CCR4, and CCR6 we identified different Th-like Treg populations (Duhen et al., 2012) in HCPS PBMCs survivors and under ANDV-GP VLPs stimuli. Our study demonstrates that memory CD4^+^ Treg from HCPS survivors present a specific phenotype. Finally, ANDV-GP-VLP modulates memory CD4^+^ Treg by downregulating CXCR3 and upregulating CCR4 surface expression.

## Results

### Higher Frequency of PD-1^+^ Memory CD4^+^ Treg on HCPS Survivors

**Figure 1:**
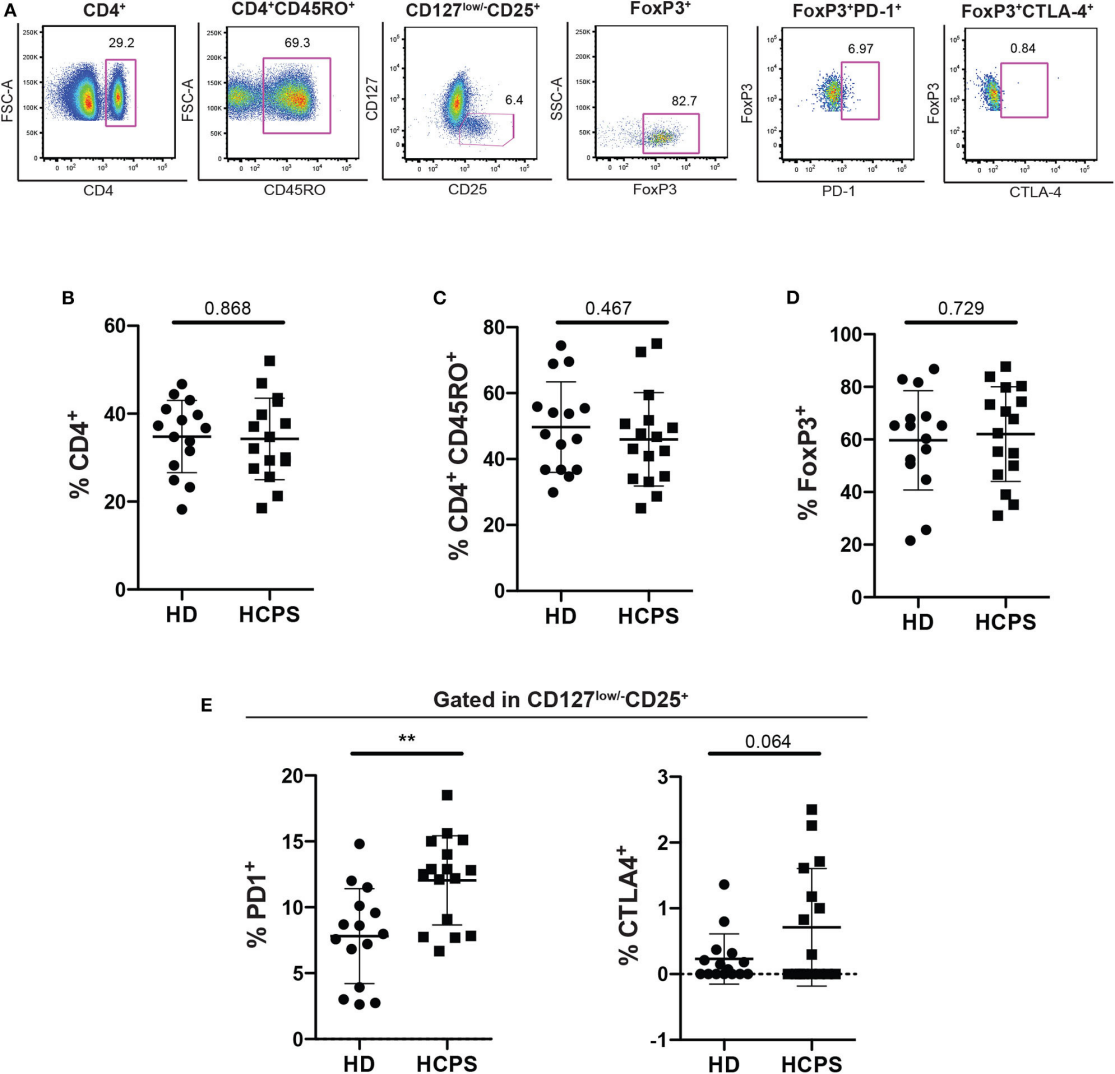
Increase frequency of memory CD4^+^ Treg PD-1^+^ on HCPS survivors. **(A)** Gating strategy of a representative sample showing CD4^+^, CD4^+^CD45RO^+^, CD127^low/−^CD25^+^, CD127^low/−^CD25^+^ FoxP3^+^, memory Treg PD1^+^, and memory Treg CTLA-4^+^ cells. **(B)** Frequency of total CD4^+^ T cells gated in live PBMC. **(C)** Frequency of memory CD4^+^ T cells CD4^+^CD45RO^+^. **(D)** Frequency of memory CD4 Treg (CD4^+^CD45RO^+^CD25^+^CD127^low/−^FoxP3^+^). **(E)** Frequency of memory Treg PD-1^+^ (left) and memory Treg CTLA-4^+^ (right). Data represented as dots (HD *n* = 15) and squares (HCPS *n* = 16), indicating the mean ± SD. **P = 0.0021 by unpaired Student's *t*-test.

To evaluate the role of circulating CD4^+^ regulatory T cells (Treg) on ANDV-survival subjects, peripheral blood mononuclear cells (PBMC) from healthy donors (HD) and ANDV convalescent subjects (HCPS) were thawed and analyzed by flow cytometry (Figure 1A). From our analysis, it was not observed differences in the frequency of CD4^+^ T cells in HCPS compared with HD (Figure 1B) or in the frequency of memory CD4^+^CD45RO^+^ T cells (Figure 1C). Also, we did not observed differences in the frequency of memory Treg (mTreg, CD4^+^CD45RO^+^CD25^+^CD127^low/−^FoxP3^+^) in HCPS with respect to HD (Figure 1D). Next, to evaluate the phenotypic state of mTreg, the expression of two suppressive proteins were investigated, PD-1 and CTLA-4 (Takahashi et al., 2000; Hui, 2019). Interestingly, the frequency of mTreg PD-1^+^ in HCPS increases significantly with respect to HD (HD: 7.81 mean ± 3.606 SD; HCPS: 12.04 mean ± 3.381 SD; *p* = 0.0021) (Figure 1E). In the case of CTLA-4 suppressor marker we did not observed changes in the frequency of mTreg expressing CTLA-4 (Figure 1E). This data suggests that PBMC of ANDV survival subjects present a specific memory CD4^+^ Treg profile. Specifically, expressed higher frequency of PD-1 in mTregs compared to healthy donors.

### HCPS Survivors Present a Decrease Th1-Like Treg Phenotype

**Figure 2:**
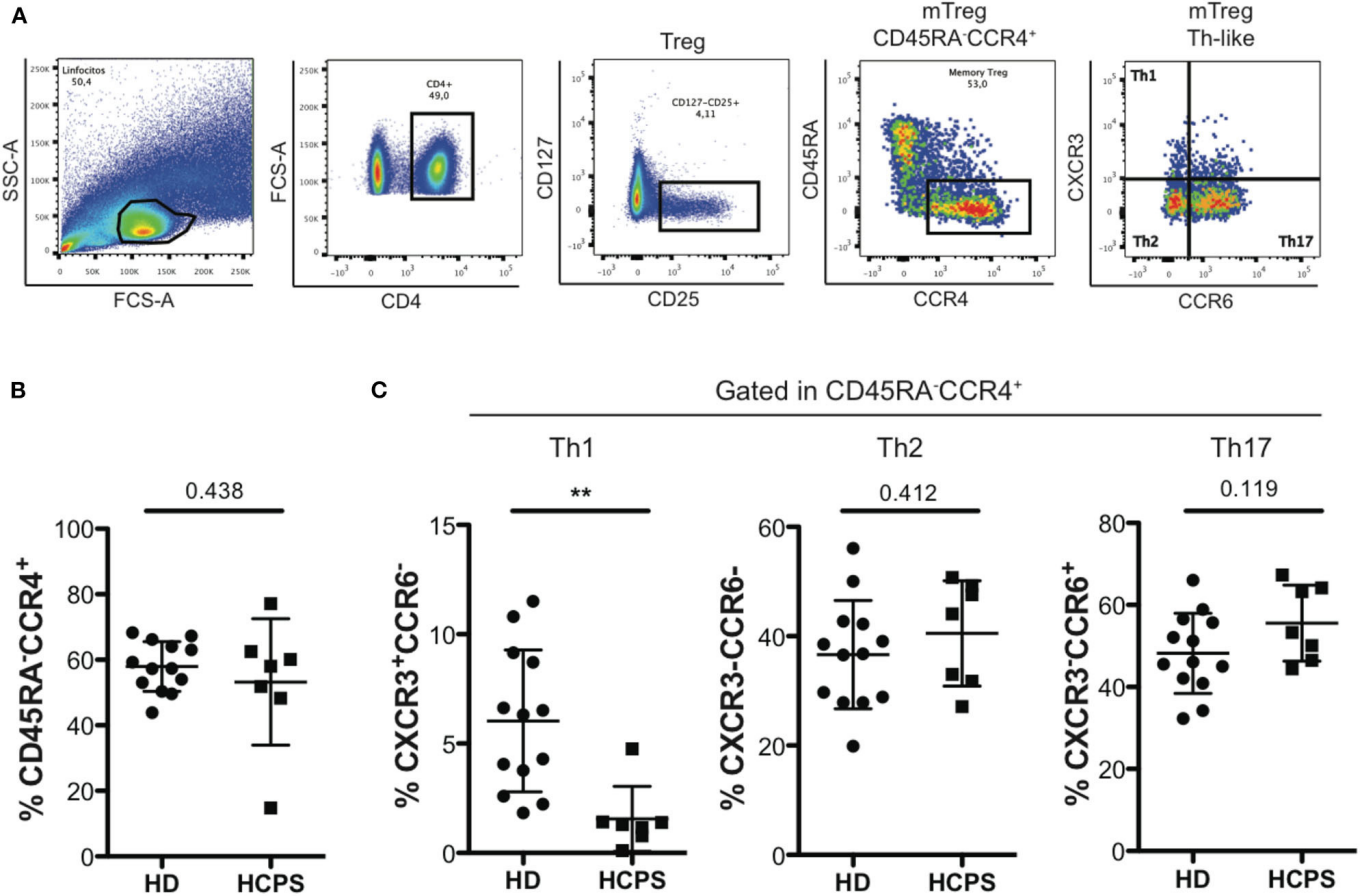
HCPS survivors present a decrease on memory Th1-like Treg. **(A)** Gating strategy of a representative sample showing the CD4^+^T cells, CD127^low/−^CD25^+^ Treg cells, memory Treg CD45RA^−^CCR4^+^, and finally the memory Th-like Treg subpopulations, specifically Th1-like Treg (CXCR3^+^CCR6^−^), Th2-like Treg (CXCR3^−^CCR6^−^), and Th17-like Treg (CXCR3^−^CCR6^+^). **(B)** Frequency of memory Treg CD45RA^−^CCR4^+^ in HD and HCPS. **(C)** Frequency of Th1-like, Th2-like, and Th17-like memory Treg in HD and HCPS. Data represented as dots (HD *n* = 13) and squares (HCPS *n* = 7), indicating the mean ± SD. **P = 0.003 by unpaired Student's *t*-test.

To further characterize the memory CD4^+^ Treg circulating compartment, mTreg Th-like subsets were determined in PBMC from HD and HCPS gating in CD25^+^CD127^low/−^CD45RA^−^CCR4^+^ follow by determination of Th1-like Treg (CXCR3^+^CCR6^−^), Th2-like Treg (CXCR3^−^CCR6^−^), and Th17-like Treg (CXCR3^−^CCR6^+^) (Figure 2A). Results shown no differences in the frequency of mTreg CD45RA^−^CCR4^+^ between HD and HCPS (Figure 2B), however, we observed a decrease in Th1-like mTreg on HCPS comparing with HD (HD: 6.033 mean ± 3.244 SD; HCPS: 1.559 mean ± 1.487 SD; *p* = 0.003). In contrast to Th1-like Tregs, an increased trend in the frequency of Th2-like mTreg population on HCPS was observed, meanwhile Th17-like Treg this population did not shown differences between HD and HCPS (Figure 2C). This result suggests that ANDV infection alters the phenotype of memory CD4^+^ Treg cells decreasing the Th1-like memory Treg population years after infection.

### ANDV-GP Induces a Th2-Like Phenotype on Memory CD4^+^ Treg Cells

**Figure 3:**
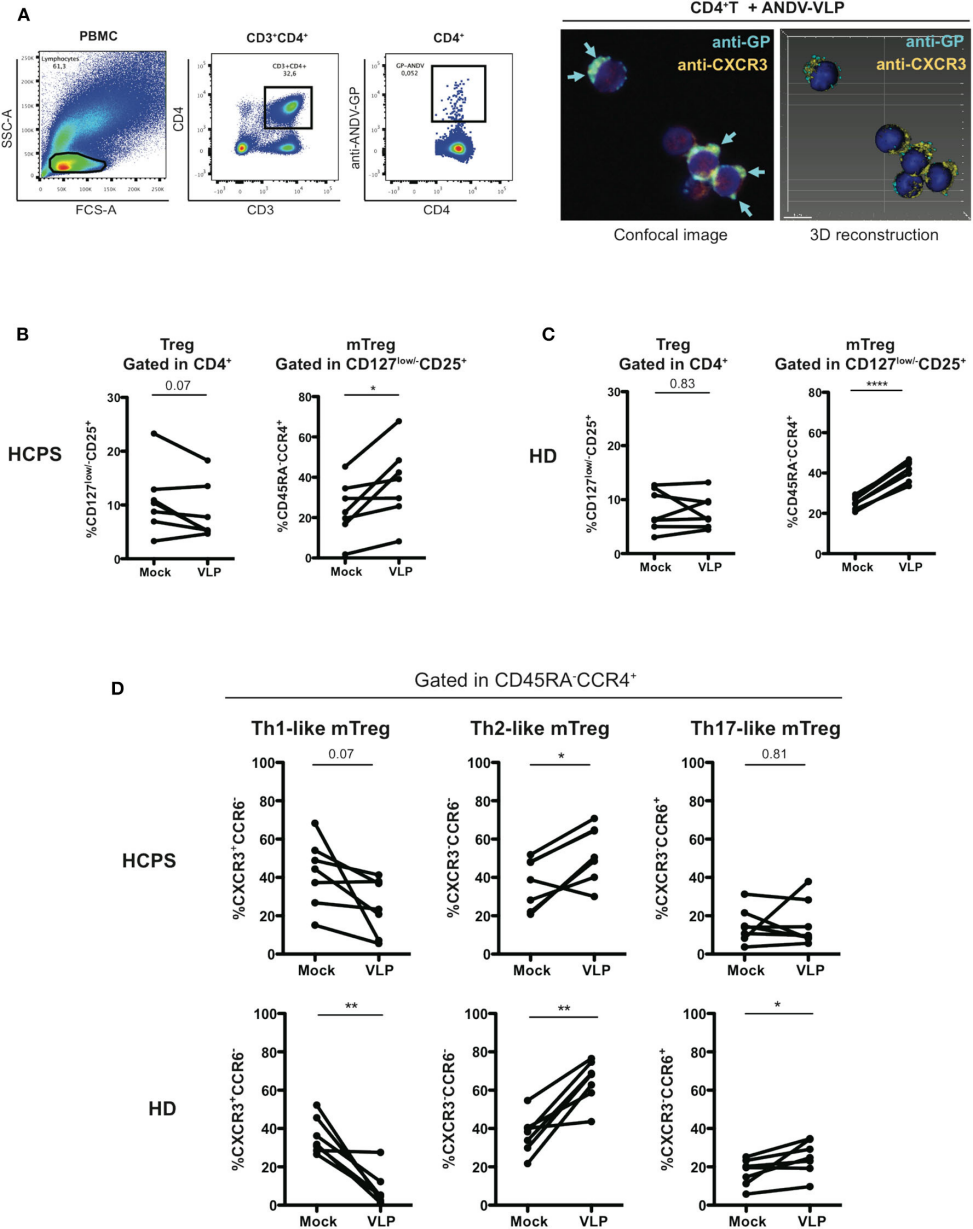
ANDV-GP virus like particles induces a Th2-like phenotype on memory CD4^+^ Treg cells. **(A)** ANDV-GP VLPs are detected in CD4^+^ T cells. PBMC were incubated with VLPs and detected by flow cytometry using an anti-ANDV-GP Qdot655 conjugated antibody (left). Purified CD4^+^ T cells were incubated with VLP and ANDV-GP was detected with anti-ANDV-GP Qdot655 conjugated antibody and visualized in CD4^+^CXCR3^+^ cells by confocal microscopy, arrows indicate the intracellular ANDV-GP staining (right). Following the same gating strategy of Figure 2 was evaluated the effect of ANDV-GP VLP on Th-like Treg frequency. **(B)** Frequency of total Treg (CD4^+^CD127^low/−^CD25^+^) in HCPS and HD PBMCs after VLP overnight stimulation follow by analysis at day 7 of cell culture. **(C)** Frequency of memory CD4^+^ Treg CD45RA^−^CCR4^+^ in HCPS and HD in mock and VLP stimulus. **(D)** Frequency of Th-like memory CD4^+^ Treg CCR4^+^ in HCPS (above panel) and HD (below panel) in mock and VLP conditions. Data for each individual is represented as a line connecting mock and VLP conditions (HD *n* = 7; HCPS *n* = 7), *P < 0.05; **P < 0.001, ****P < 0.0001 by paired Student's *t*-test.

To determine whether ANDV-GP could affect directly CD4^+^ T cells, first, PBMC were incubated with ANDV-GP virus like particles (VLP) and using intracellular staining and flow cytometry we specifically detected ANDV-GP in CD4^+^ cells, to prove this finding purified CD4^+^ T cells were incubated with VLP observing the internalization of ANDV-GP by confocal microscopy (Figure 3A), these data shown that ANDV-GP can be internalized by CD4^+^ T cells potentially impacting this cell population.

Next, to examine the hypothesis that ANDV-GP could induce the phenotype observed in the Treg profile of HCPS survivor subjects, PBMC from HD and HCPS subjects were stimulated with ANDV-GP VLP for 16 h or mock treated and analyzed by flow cytometry after 7 days. In HCPS PBMCs, VLP stimuli did not affect the frequency of total Treg (Figure 3B) but lead to an increase on the frequency of mTreg CCR4^+^ (Mock: 24.31 mean ± 13.91 SD; VLP: 37.31 mean ± 18.81 SD; *p* = 0.0211) (Figure 3B). The same trend was observed when HD PBMCs were analyzed; VLP stimuli did not change the frequency of total Treg but induced increase in the frequency of mTreg CCR4^+^ (Mock: 25.26 mean ± 3.146 SD; VLP: 41.13 mean ± 5.074 SD, *p* < 0.0001) (Figure 3C). Noteworthy, when was analyzed the Th-like subsets of mTreg CCR4^+^ in HCPS (gated in CD45RA^−^CCR4^+^), we observed that the VLPs induced a decrease in the frequency of Th1-like mTreg near to 2-fold in comparison with un-stimulated condition (Mock: 42.13 mean ± 17.65 SD; VLP: 24.7 mean ± 14.68 SD; *p* = 0.0701) which is consistent with the decrease in CXCR3^+^ Th1-like observed in HCPS convalescent subjects (Figure 2C) and a significant increase in the frequency of Th2-like Treg on HCPS (Mock: 36.83 mean ± 13.13 SD; VLP: 52.71 mean ± 14.75 SD; *p* = 0.0152), without changes in Th17-like mTreg (Figure 3D). Regarding the effect of VLP on HD over the Th-like Treg populations, we observed an overall redistribution in Th-like mTreg population, with an important decrease in the Th-1 like mTreg frequency, near to 4-fold in comparison with mock condition (Mock: 35.93 mean ± 9.545 SD; VLP: 8.346 mean ± 9.158 SD; *p* = 0.0033) and a significant increase on the Th-2 like mTreg (Mock: 36.99 mean ± 10.23 SD; VLP: 64.73 mean ± 11.2 SD; *p* = 0.0017), in line with the findings in the HCPS cohort after VLP stimuli. Additionally, an increase in Th-17 like mTreg population was present in HD population (Mock: 17.14 mean ± 6.938 SD; VLP: 25 mean ± 8.874 SD, *p* = 0.0357) (Figure 3D). To further evaluate the effect of the ANDV-GP, we determined the frequency of the suppression markers PD-1 and CTLA-4 on mTreg CD127^low/−^CD25^+^FoxP3^+^ after VLP stimuli in HCPS and HD PBMCs. We did not find differences in the frequency of mTreg in HCPS or in HD. The same tendency was observed when analyzed PD-1 and CTLA-4 in HCPS and HD (Supplementary Figure 1).

This data suggest that VLP can modify the phenotype of CD4^+^ Tregs not only in cells from HD, but also in HCPS patients, promoting a disbalance between Th1 and Th2 lineages. This could suggest that hantavirus impair the antiviral response by affecting the proinflammatory Th1 response and all the mechanisms supported by this subset. On the other hand, the fact that ANDV-GP induced a higher Th2-like Tregs could also suggest that a potent regulatory response is also relevant for HCPS survival.

### ANDV-GP Induces Th1/Th2 Cytokine Expression on Memory CD4^+^ T Cells

**Figure 4:**
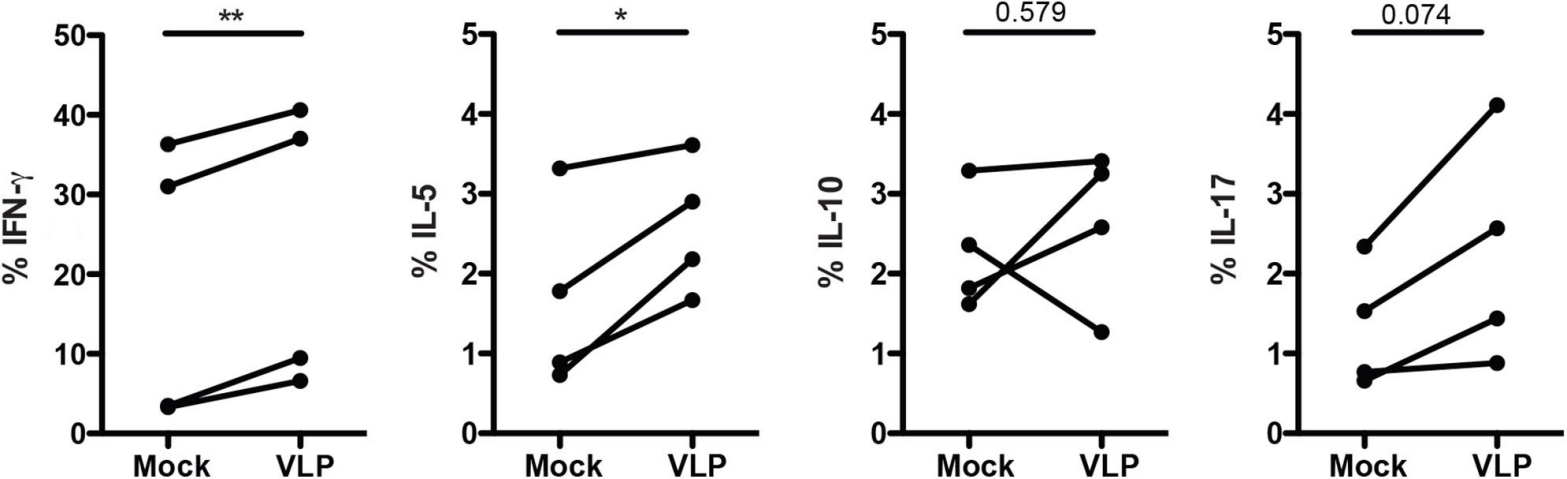
ANDV-GP induces Th1/Th2 cytokine expression on memory CD4^+^ T cells. Frequency of memory CD4^+^ T cells expressing the following cytokines (IFNγ, IL-5, IL-10, and IL-17) in HD stimulated with VLP in presence of anti-CD3/CD28 beads or mock as control after 3 days in cell culture (*n* = 4). Each line represents a subject sample. *P < 0.05; **P < 0.001, by paired Student's *t*-test.

Since ANDV-GP VLPs could modulate mTreg phenotype in both HD and HCPS, we evaluated the effect of ANDV-GP on cytokine expression. Isolated memory CD4^+^ T cells from healthy donors were stimulated with VLP by 16 h in presence of anti-CD3/anti-CD28 beads and intracellular cytokine was determined (Figure 4). The results indicate a significant increase in the frequency of memory CD4^+^ T cells expressing IFNγ (Mock: 18.53 mean ± 17.6 SD; VLP: 23.42 mean ± 17.86 SD; *p* = 0.0052) and IL-5 (Mock: 1.68 mean ± 1.187 SD, VLP: 2.590 mean ± 0.8468 SD, *p* = 0.0350) (Figure 4) related to Th1 and Th2 profile, respectively, in line with previous reports in ANDV affected patients (Borges et al., 2008), not significant differences it was found in the frequency of memory CD4^+^ T cells expressing IL-10 where two subject increased, one maintain and another decreased their frequency of cells expressing IL-10, suggesting that Treg cytokines profile did not present an specific pattern after ANDV-GP encounter. In the case of IL-17 cell frequency was increased but the change was not significant (Figure 4).

### ANDV-GP Modulate CD4^+^ Treg Phenotype by Down Regulating CXCR3

**Figure 5:**
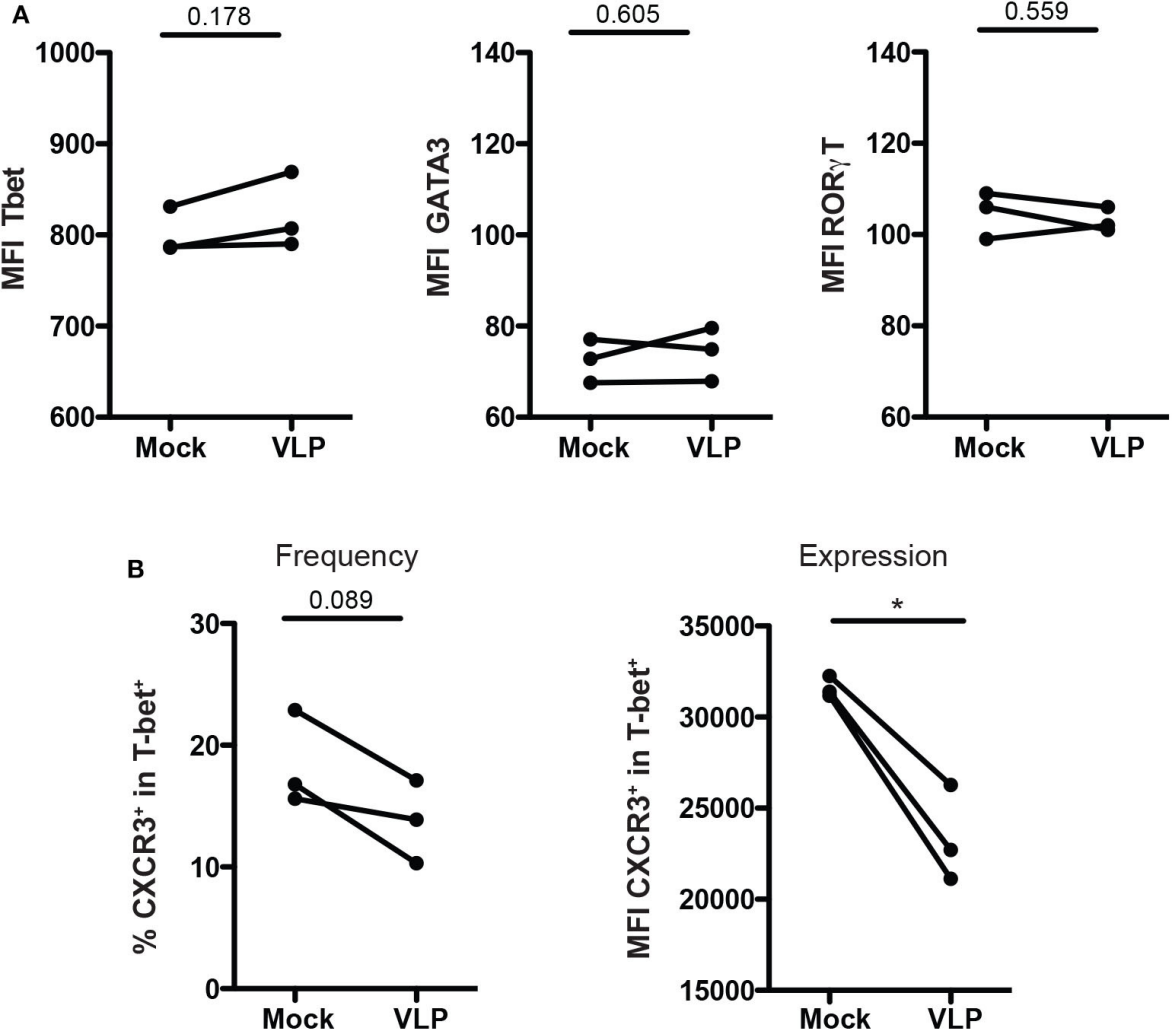
ANDV-GP modulates CD4^+^ Treg phenotype by down regulating CXCR3. Three healthy donors were used to isolate CD4^+^ Treg from PBMCs, stimulated with VLP or no-treated (mock) and analyzed after 3 days in cell culture. **(A)** Mean fluorescence intensity (MFI) of T-bet, GATA3, and RORγt that allows the detection of Th1-like Treg, Th2-like Treg, and Th17-like Treg, respectively. **(B)** Frequency and expression measured by MFI of CXCR3 on the T-bet^+^ CD4^+^Treg population. Each line represents a subject sample (*n* = 3). *P < 0.05, by paired Student's *t*-test.

To determine whether the downregulation of Th1-like Tregs and upregulation of Th2-like Tregs cells are due to change of T helper master regulator transcription factors, we evaluate the expression of T-bet, GATA3, and RORγT in isolated Treg cells culture (CD4^+^CD25^+^CD127^low/−^) upon ANDV-GP VLP stimuli from three different healthy donors. The results shown that Treg cells do not change the expression of transcription factors T-bet (Mock: 801.3 mean ± 25.7 SD, VLP: 822 mean ± 41.58 SD; *p* = 0.1775), GATA3 (Mock: 72.5 mean ± 4.757 SD, VLP: 74.13 mean ± 5.888 SD; *p* = 0.6045) or RORγT (Mock: 104.7 mean ± 5.132 SD, VLP: 103 mean ± 2.646 SD; *p* = 0.5598) after ANDV-GP VLP treatment (Figure 5A), confirming that the change on phenotype of Treg is not due to shift in expression of transcription factors.

Next, due to the decrease of Th-1 like population in mTreg cells in HCPS subjects, and also in mTreg cells from HD and HCPS treated with ANDV-GP VLPs, the frequency and expression of CXCR3 was evaluated in purified CD4^+^CD25^+^CD127^low/−^T-bet^+^ T cell cultures (Figure 5).

The results showed a reduction in frequency of Treg CXCR3^+^ (Mock: 18.43 mean ± 3.915 SD, VLP: 13.77 mean ± 3.402 SD; *p* = 0.0893) (Figure 5B), but also a significant down regulation of CXCR3 expression levels measured as MFI in CD4^+^CD25^+^CD127^low/−^T-bet^+^ cells (Mock: 31643 mean ± 574.1 SD, VLP: 24396 mean ± 2822 SD; *p* = 0.0356) (Figure 5B). These results, suggest that the profile observed after VLP stimulation involved changes in chemokine receptor level but not in T-bet expression.

## Discussion

Several studies suggest that the immune response could be an important part in the pathology observed in HCPS (Mori et al., 1999; Khaiboullina et al., 2017). However, it is not clear whether the strong immune response is only the consequence of a severe infection or a key player on HCPS pathogenesis. In this study we tested the hypothesize that ANDV could establish specific features potentially tracked in memory CD4 Treg compartment of HCPS survivors, which are responsible to maintain tolerance and suppress inflammatory immune response (Sakaguchi et al., 2008).

In this context, several investigations have suggested a role of Treg on hantavirus persistence in rodents, speculating that Treg cells play an important role in limiting the immunopathology (Easterbrook et al., 2007; Schountz et al., 2007; Easterbrook and Klein, 2008). On the other hand, in Syrian hamsters infected with a lethal dose of ANDV, a strong suppression of Treg responses was observed and hypothesized to be the basis of an aberrant immune activation (Safronetz et al., 2011). Nevertheless, it is not well understood the role played for CD4^+^ Treg in human ANDV infection.

We focus our analysis in the CD4^+^ Treg cells of HCPS survivors. Our results show no changes in the frequency of memory CD4^+^ T cells or in the memory CD4^+^ Treg cells. However, we found significant differences in the phenotype of CD4^+^ Treg cells of HCPS compare to HD cohort.

Our first hypothesis was that HCPS survivors from ANDV infection present a specific CD4^+^ Treg signature that may be involved in control the strong immune response during HCPS, therefore we analyze the frequency of memory CD4^+^ T cells and we seek for specific pattern on CD4^+^ Treg function. We analyzed immune regulatory suppressor marker CTLA-4 and PD-1. The frequency of CTLA-4 on memory CD4^+^ Treg cells showed no differences in comparison HCPS with HD. Interestingly, PD-1 an important immune regulatory suppressor marker was found increased in the HCPS cohort. Specifically, HCPS survivor's expressed higher frequency of PD-1 in memory CD4^+^ Treg cells compared to HD, suggesting an immunosuppressive phenotype on these cells (Figure 1). This result differs with previous longitudinal study performed on a cohort of HFRS infected with PUUV (Lindgren et al., 2011) which showed that PD1^+^CD4^+^ T cells increased during the acute phase of infection and then reduced rapidly during convalescent phase. Besides, a new report showed that patients infected with hantavirus up regulated PD ligand 1 (PD-L1) in serum sample (Raftery et al., 2018). PD-L1 expression can induce an increase of CD4^+^ Treg cells and PD-1 is a functional marker for CD4 Treg suppressor activity. Indeed, the induction of PD-1 in hantavirus infection could be of relevance since endothelial cells, the main target cells of hantavirus, upregulate PD-L1 under stress conditions (Eppihimer et al., 2002; Mueller et al., 2010). In this context, we tested the idea that ANDV-GP could be responsible to induce the expression of PD-1, however no significant differences in CD4^+^ Treg cells were observed after ANDV-GP VLPs stimulation (Supplementary Figure 1). These results could be explained by the fact that in our experimental set up we are using VLP lacking nucleoprotein (NP) and any other viral protein and /or determinant, since our focus of study is elucidating the effect of ANDV-GP on CD4^+^ Treg cell response.

Because we found no difference in the frequency of memory CD4^+^ Treg cells in HCPS survivors, we next focus our analysis in the CD4^+^ Treg subsets phenotype. Several studies have reported that CD4^+^ Treg cells use differential transcription program to regulate Th1, Th2, and Th17 responses, and that these are associated with the expression of specific transcription factors (Chaudhry et al., 2009; Koch et al., 2009; Zheng et al., 2009; Duhen et al., 2012). Indeed, CD4^+^ Treg cells differentiate into specialized subsets during the immune responses and this is essential for appropriate regulation of different Th cell population (Duhen et al., 2012). Searching for a specific phenotype imprinted in CD4^+^ Treg cells and based on differential expression of chemokine receptors CCR4, CXCR3, and CCR6 we described the subsets of CD4^+^ Treg cells present in HCPS survivors. Surprisingly we found a diminished frequency of Th1-like Treg sub-population defined as a CCR6^−^CXCR3^+^CCR4^+^CD45RA^−^CD127^low/−^CD25^+^CD4^+^ T cells (Figure 2). This is particularly interesting since during infection Th1 CD4^+^ T cell response are a primary response fundamental to control viral infection and Th and Treg cells differentiate in parallel phenotypes in response to inflammatory signals (Duhen et al., 2012). To better understand the implication of this finding we tested the hypothesis that ANDV-GP could be responsible for this established phenotype. Interestingly, we found that CD4^+^ T cells are able to internalize ANDV-GP VLP, potentially impacting this population (Figure 3). We found that ANDV-GP VLPs down regulated frequency of Th1-like Treg and up regulate frequency of Th2-like Treg in cells obtained from healthy donors (first antigen encounter) or HCPS survivor cells (previously exposed to the virus, recall experiment) (Figure 3). This result suggest that ANDV-GP could alter the phenotype of memory CD4^+^ Treg cells, and that signature could be preserved even years after resolution of the infection.

Next, we evaluate the idea that this phenotype could be regulated via transcription factor. The analysis performed upon ANDV-GP stimuli did not change the expression of Th1 transcription factor T-bet, Th2 transcription factor GATA3 or Th17 transcription RORγT in CD4^+^ Treg cells indicating a different mechanism (Figure 5). However, it was confirmed that CD4^+^ Treg expressing the transcription factor T-bet down regulated CXCR3 expression (Figure 5). Th1 transcription factor T-bet is normally associated with synthesis of inflammatory cytokines such as IFNγ (Oh and Hwang, 2014). In our study, IFNγ was over expressed in memory CD4^+^ T cells treated with ANDV-GP VLPs suggesting a polarized Th1 response. However, the frequency of memory Treg cells expressing CXCR3 were significantly lower in HCPS survivors compare to HD. Additionally, CXCR3 was down regulated and CCR4 up regulated in memory CD4^+^ Treg exposed to ANDV-GP VLPs, this suggest that ANDV-GP could specifically act over CXCR3 in CD4^+^ Treg cells potentially altering their ability to migrate to the site of infection (Groom and Luster, 2011; Kallies and Good-Jacobson, 2017). Other important chemokine receptor is CCR4 that has been shown in animal models to be required for Treg recruitment to non-lymphoid tissues such as lung and involved in attenuation of lung inflammation (Sather et al., 2007; Faustino et al., 2013). For sure, more studies are necessary to further characterize this phenotype and determine the immune cells populations involved in this response. Longitudinal studies will be needed to understand the specific Th1/Th2 CD4^+^ Treg cell response during ANDV infection and their impact in immunopathogenesis.

## Materials and Methods

### Human Samples

HCPS survivors (*n* = 16) were enrolled between 1 and 14 years after hospitalization. Cohort characteristics are summarized in Supplementary Table 1. Fifteen healthy donors (HD) without story of HCPS were included; group characteristics are summarized in Supplementary Table 2. All participants included were Chilean citizens and volunteered participate in the study after signed informed consent in accordance with approval of Institutional Review Boards protocols (Ministry of Health, Valdivia Health Service, protocol number 456). Specifically, 50 ml of peripheral venous blood were obtained using EDTA tubes. PBMCs were isolated by Ficoll-Hypaque density-gradient centrifugation and store in liquid nitrogen. Fresh PBMCs were used for intracellular cytokine detection. To control previous exposure to ANDV hantavirus infection, antibodies against ANDV-GP was evaluated by ELISA in serum samples (Supplementary Figure 2).

### Enzyme Linked Immunosorbent Assay (ELISA)

To detect serum antibodies against ANDV-GP, an ELISA using pseudoviral particles as previously described was performed (Garrido et al., 2018). Briefly, costar plates (Corning) were coated with viral particles pseudotyped with ANDV-GP (ANDV-pv) or VSV-G (VSV-G-pv) as control of a non-related viral envelope. Pseudovirus was concentrated by a 20% sucrose cushion and ultracentrifuged at 145,000 × g and normalized using a HIV p24 ELISA (Sino Biological). 10 pg/ml of pseudovirus were used to coat plates, after washing with PBS-0.05% Tween-20 (PBS-T) and blocking, serum samples (1/250 dilution) were added by 1 h at 37°C. Following washes, plates were incubated with peroxidase-labeled goat anti-human IgG+IgM (1/5,000) (Jackson ImmunoResearch). Finally, plates were developed using 3,3′5,5′ tetramethylbenzidine (TMB) (eBioscience), and sulfuric acid 2N was used for stopping reaction. Optical density (OD) was measured at 450 nm.

### Virus Like Particle (VLP) Production

VLPs were produced by transfecting 293T cells with ANDV-GP (Chile-9717869) using calcium phosphate transfection method (Pear et al., 1993). Sixteen hours after transfection, the medium was replaced with Dulbecco's modified Eagle's medium (DMEM) (Corning) supplemented with 10% cosmic calf serum (CCS), L-glutamine, and antibiotics. After 72 h of transfection, ANDV-GP VLPs were collected, filtered through a 0.45 μm filter (Merck) and concentrated by ultracentrifugation at 145,000 × g in a 20% sucrose cushion. Production of VLPs was evaluated by western blot using a specific monoclonal antibody against ANDV-GP (Garrido et al., 2018). Specifically, Gn/Gc recombinant protein (kindly provided by Dr. Rey and Dr. Guardado from Institut Pasteur, France), ANDV-GP VLP, and viral particles pseudotyped with ANDV-GP (ANDV-pv) were loaded into a non-denaturing 8% polyacrylamide gel for electrophoresis. After a 16 h transfer, PVDF membrane was blocked with 5% non-fat milk, incubated with anti-ANDV-GP monoclonal antibody (Ichor Biologics) and goat anti-human IgG HRP secondary antibody (Jackson ImmunoResearch). Images were obtained with 10PXi gel documentation system (Supplementary Figure 3).

In addition, ANDV-GP concentration was tested by ELISA sandwich using Gn/Gc recombinant protein as standard curve and monoclonal antibodies against ANDV-GP provided by Ichor Biologics (Supplementary Figure 3). The experiments were performed using 100μl of VLP (2 μg/ml).

### Detection of VLP in PBMC and CD4^+^ Cells

For flow cytometry VLP detection, PBMCs from HD were incubated for 2 h at 37°C with VLP, washed and stained with anti-CD3 and anti-CD4 antibodies, follow by intracellular staining with Qdot-655 conjugated anti-ANDV-GP monoclonal antibody (Garrido et al., 2018).

For immunofluorescence assay, purified CD4^+^ T cells from HD were incubated with ANDV-GP VLP for 2 h at 37°C. After three washes with 3% PBS-BSA, they were fixed with 0.5% PFA-3% BSA in PBS for 20 min. Then, cells were incubated with Hoechst stain, Qdot-655 conjugated anti-ANDV-GP monoclonal antibody and anti CXCR3-APC antibody in 3%BSA-0.1% Tween-20 for 1 h. Confocal imaging and 3D reconstruction were obtained at CMA Bío-Bío facility.

### Phenotypic Analysis of Memory Treg, Suppression Markers, and Subsets From Peripheral Blood

According sample availability, PBMC from HCPS and HD were thawed and cultured in 48 wells plate using RPMI-1640 medium (Corning) supplemented with 10% fetal bovine serum (FBS), 1% antibiotics, and L-glutamine (Corning) and 50 UI/mL of IL-2 (Sigma-Aldrich) (complete media). After 1 day 500,000 PBMCs were challenged overnight with GP-ANDV VLP, cells without stimuli for each individual was included as control (mock). mTreg were stained in baseline conditions and after 1 week. First, Fixable viability stain AF780 (BD Biosciences) was diluted 1/10,000 in PBS 1X and incubated at room temperature in the dark for 10 min. Later, to determine the mTreg population, antibodies were mixed in PBS-BSA 0.5% and incubated for 30 min at 4°C in the dark, the following antibodies were used: anti-CD4-PerCPVio700 (Miltenyi Biotec), anti-CD45RO-PeVio770 (Miltenyi Biotec), anti-CD25-BV650 (BD Biosciences), anti-CD127-PE, anti PD-1-FITC, and CTLA-4-BV421 (BD Biosciences). After washes, anti-FoxP3-Alexa Fluor 647 (BD Biosciences) was added and transcription factor buffer set was used following the manufacturer instructions (BD Biosciences). For subsets of mTreg, HCPS, and HD were included according to the sample availability. PBMCs were stained using the following antibodies: anti-CD4-BV421 (eBiosciences), anti-CD25-PECy7 (eBiosciences), anti-CD127-PerCPCy5.5 (eBiosciences), CD45RA-APC-Cy7 (BioLegend), anti CCR4-APC (BioLegend), anti-CXCR3-AF488 (BioLegend), and anti-CCR6-BV650 (BioLegend) (Halim et al., 2017). Cells were collected in a BD LSR-Fortessa-X20 and files analyzed using FlowJo (TreeStar). Gates were set based in single color stained and fluorescence minus one control.

### Memory CD4^+^T Cell Isolation, Transcription Factors, and Cytokine Analysis

Intracellular staining of FoxP3, IFNγ, IL-5, IL-17, and IL-10 was performed in CD4^+^ T cells from HD purified by negative selection using CD4^+^CD45RO^+^ microbeads kit following the protocol of manufacturer (Miltenyi Biotec). Memory T CD4^+^ cells were sorted in FACSAria III obtaining more than 98% purity. Cells were recovered overnight with RPMI complete media and the next day 1 × 10^6^ memory CD4^+^ T cells were challenged with ANDV-GP VLP in presence of 2.5 μL of anti-CD3/CD28 beads (ThermoFisher Scientific), also, an anti-CD3/CD28 condition was included and without stimuli was used as control (mock). After 3 days, cells were stimulated with 50 ng/ml phorbol 12-myristate 13-acetate (PMA) and 1 μg/ml ionomycin (Sigma Aldrich) in presence of 1 μL brefeldin A (Biolegend) for 5 h. Later, cells were stained with fixable viability stain AF780 and fixed/permeabilized with transcription factor buffer set following manufacturer instructions (BD Biosciences). Intracellular staining was performed using the following antibodies: anti-IFNγ-FITC (Biolegend), anti-IL-5-PE (BioLegend), anti-FoxP3-eFluor450 or anti-IL-10-PE, and anti-IL-17-FITC (eBioscience).

### Treg Isolation and Staining

PBMC from HD were used for Treg purification using the CD4^+^CD25^+^ regulatory T cell isolation kit (Miltenyi Biotec). Follow by staining with CD4-FITC, CD25-PeCy7, and CD127-PerCP Cy5.5 (BioLegend) and FACSAria III sorting obtaining ~95% purity of CD4^+^CD25^+^CD127^low/−^ Treg cells. 10^5^ cells were recovered by 24 h with RPMI complete media and then stimulated with ANDV-GP VLP including a mock (without stimuli) overnight. After 3 days, cells were surface stained with CXCR3-FITC follow by fixable viability stain FVS780 and intracellular staining with the following antibodies: T-bet-PerCP, GATA3-APC, and RORγT-BV421 (BioLegend) using the transcription factor buffer set according manufacturer protocol (BD Biosciences). Finally, cells were analyzed by flow cytometry.

### Statistics

Statistical analysis was performance using PRISM 6.0 software (GraphPad) and expressed as mean ± SD. For differences in cell frequency between HD and HCPS groups, unpaired student *t*-test was applied according to Shapiro-Wilk normality test. While, paired student *t*-test was used for comparison between mock and ANDV-GP VLP stimuli condition for each individual. Differences were considered statistically significant when *p*-value was < 0.05. Significant values are indicated as asterisk as follow: ^*^p < 0.05, ^**^p < 0.01, ^***^p < 0.001, ^****^p < 0.0001.

## Data Availability Statement

The datasets generated for this study are available on request to the corresponding author (Maria Ines Barria).

## Ethics Statement

All participants included were Chilean citizens and volunteered to participate in the study after providing signed informed consent in accordance with the approval of the Institutional Review Boards protocols (Ministry of Health, Valdivia Health Service, protocol number 456). The patients/participants provided their written informed consent to participate in this study.

## Author Contributions

FS and EN-L performed experiments, analyzed data, and review the manuscript. FF-V performed experiments. JG, RA, and MB discussed, wrote, and reviewed the manuscript. MC, RR, MR, CC, and LF in charge of cohort recruitment and clinical review of cases. All authors approved the manuscript.

## Conflict of Interest

JG and RA were partially employed by the company Ichor Biologics. The remaining authors declare that the research was conducted in the absence of any commercial or financial relationships that could be construed as a potential conflict of interest.
