# Supplementary material for: Differential CD4 T Regulatory Cell Phenotype Induced by Andes Hantavirus Glycoprotein
Source: Front Cell Infect Microbiol. 2020 Aug 25;10:430. doi: 10.3389/fcimb.2020.00430 (PMC7477076; doi:10.3389/fcimb.2020.00430)
Supplement: Supplementary file 1 [file Table_1.DOCX]

**Supplementary Table 1.** Characteristics of the HCPS cohort, indicating age, sex, severity of HCPS, residence and working area. In severity of HCPS mild was considered as subjects that received oxygen and severe as those whom required vasoactive drug therapy and/or mechanical ventilation at time of hospitalization, n=16.

| Characteristic | Value |
| --- | --- |
| Age (mean ± SD) | 47 ± 17.6 |
| Years after HCPS | 5.4 ± 4.0 |
| Sex |  |
| Female | 6 |
| Male | 10 |
| Severity of HCPS |  |
| Mild | 7 |
| Severe | 9 |
| Residence |  |
| Urban | 5 |
| Rural | 11 |
| Worked area |  |
| Urban | 6 |
| Rural | 9 |
| No data | 1 |

**Supplementary Table 2.** Characteristics of the healthy donors, indicating age, sex, clinical antecedents, risk factors for hantavirus infection and residence. Clinical antecedents refer to cardio and/or respiratory antecedents and risk factors for hantavirus infection refers to perform summer activities in rural areas such as camping and trekking, n=15.

| Characteristic | Value |
| --- | --- |
| Age (mean ± SD) | 37 ± 13.3 |
| Sex |  |
| Female | 8 |
| Male | 7 |
| Clinical antecedents^a^ |  |
| Yes | 2 |
| No | 13 |
| Risk factors for hantavirus infection^b^ |  |
| Yes | 5 |
| No | 10 |
| Residence |  |
| Urban | 15 |
| Rural | 0 |

^a^ Cardio and/or respiratory antecedents.

^b^ Risk factors related with summer activities in rural areas such as camping and trekking.
