# Supplementary figures and images for: Differential CD4 T Regulatory Cell Phenotype Induced by Andes Hantavirus Glycoprotein
Source: Front Cell Infect Microbiol. 2020 Aug 25;10:430. doi: 10.3389/fcimb.2020.00430 (PMC7477076; doi:10.3389/fcimb.2020.00430)

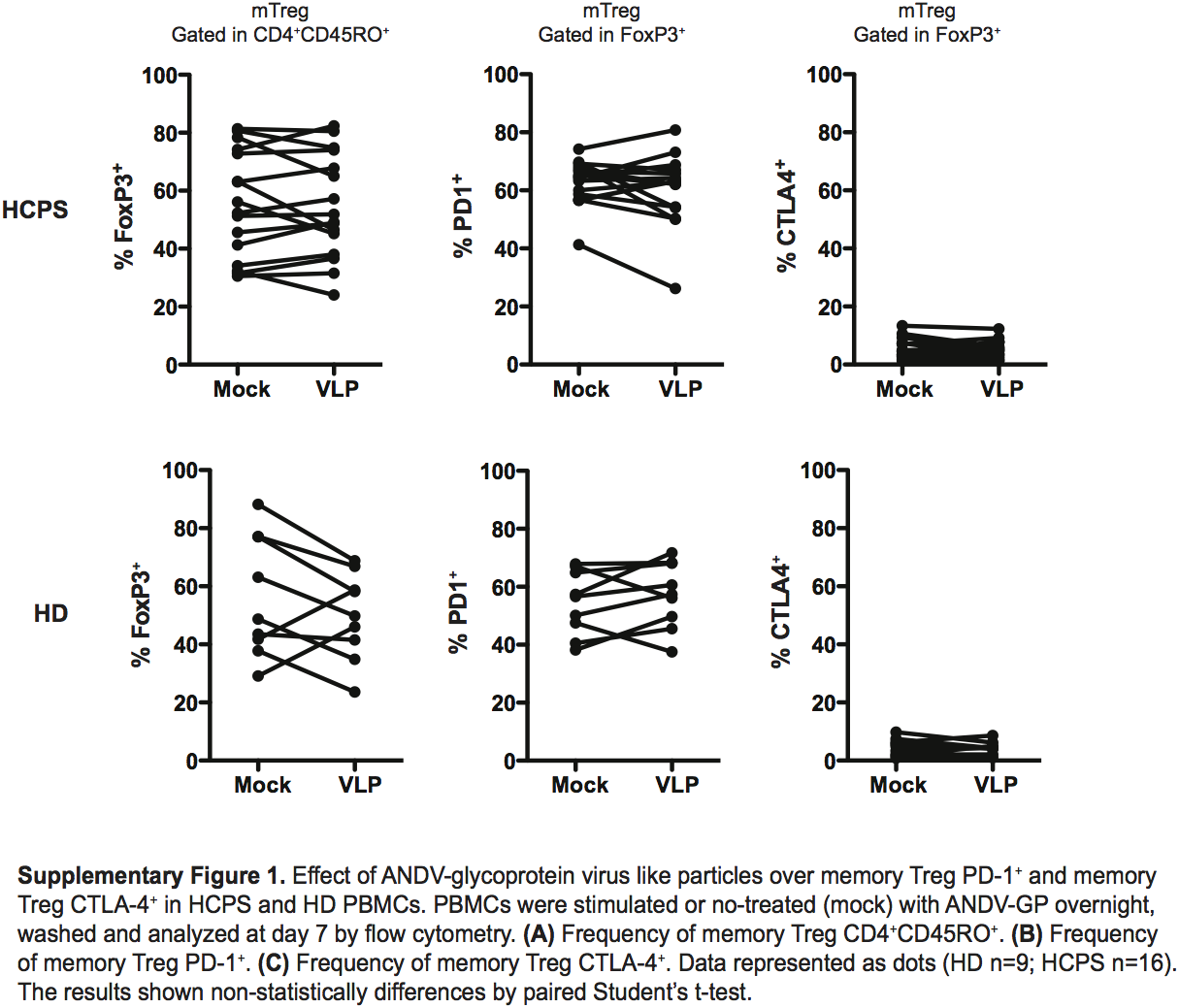

Supplement: Supplementary file 2 [file Image_1.TIFF]

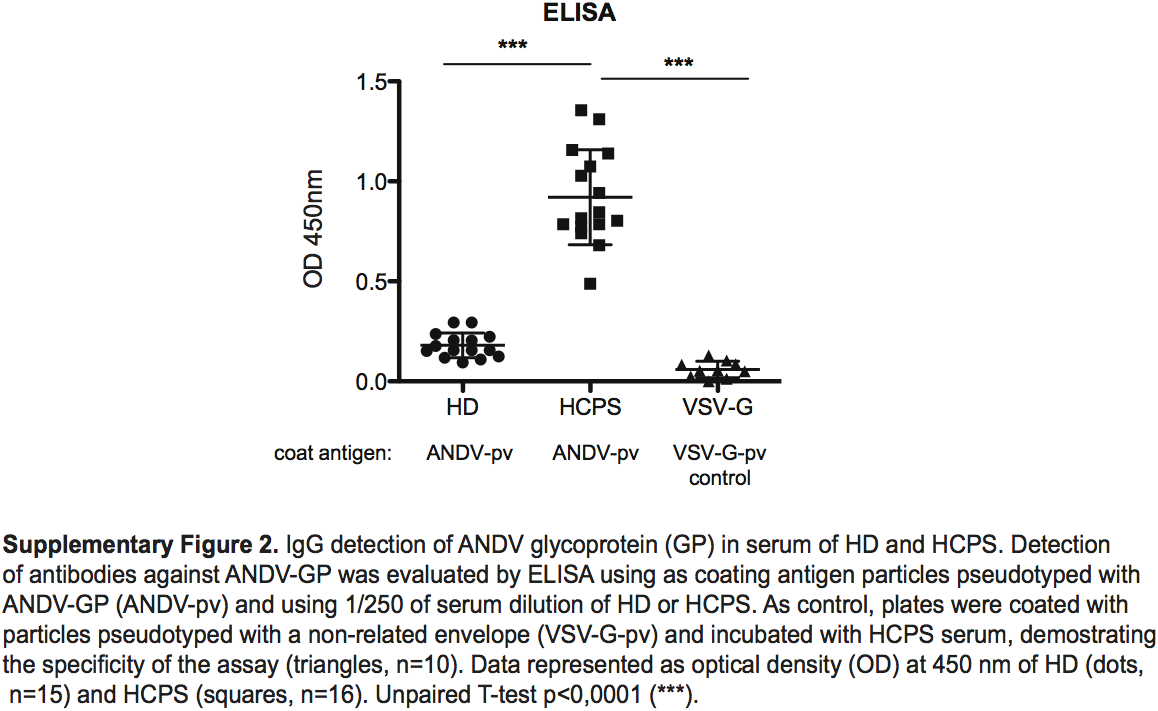

Supplement: Supplementary file 3 [file Image_2.TIFF]

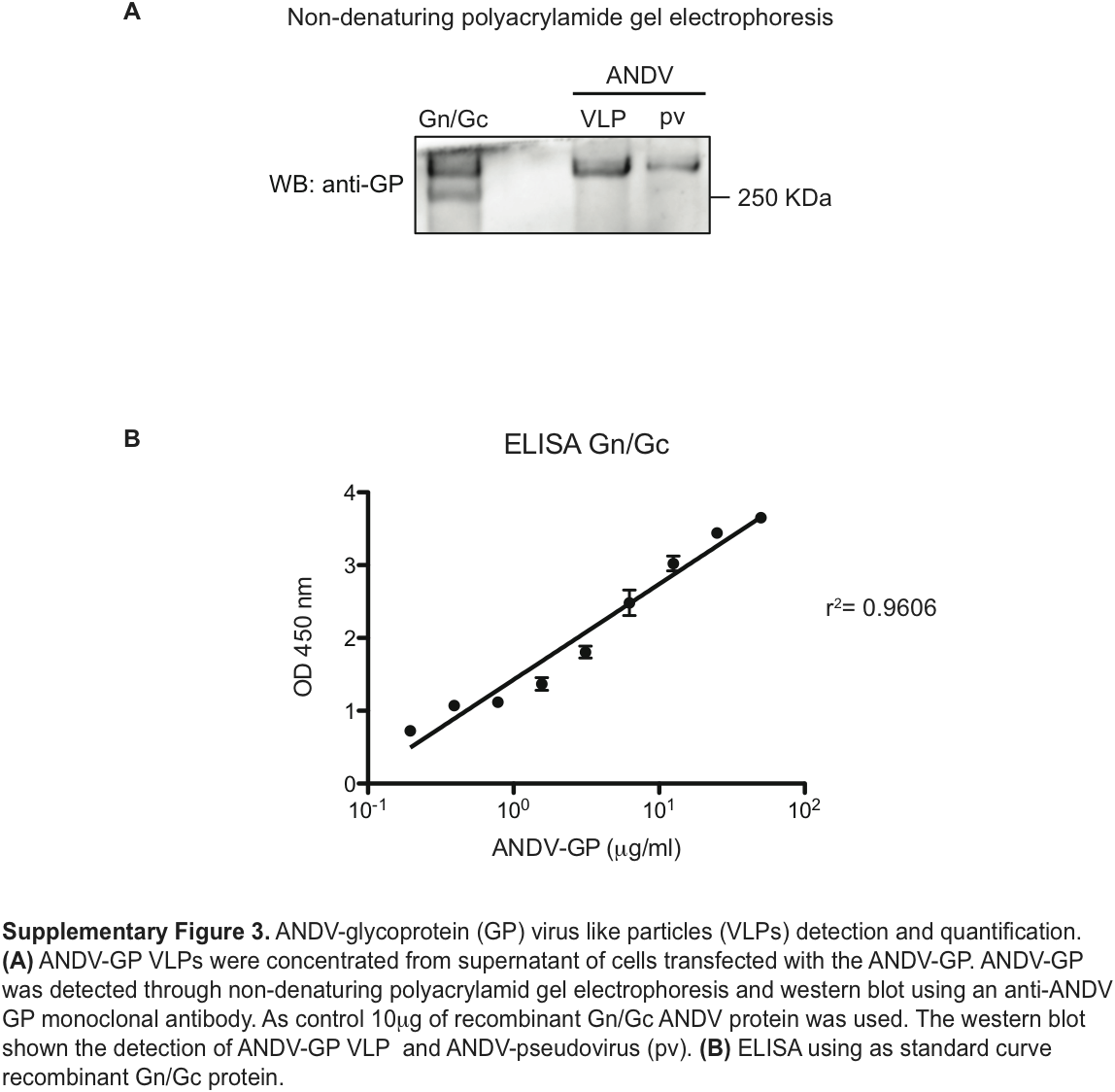

Supplement: Supplementary file 4 [file Image_3.TIFF]
